# Inferring the locomotor ecology of two of the oldest fossil squirrels: influence of operationalization, trait, body size and machine learning method

**DOI:** 10.1098/rspb.2024.0743

**Published:** 2024-11-13

**Authors:** Jan Wölfer, Lionel Hautier

**Affiliations:** ^1^ Humboldt-Universität zu Berlin, Philippstraße 13, Berlin, Germany; ^2^ Institut des Sciences de l’Évolution de Montpellier, UMR 5554, Univ de Montpellier, CNRS, IRD, Montpellier, Cedex 5, France

**Keywords:** femur, geometric morphometrics, *Palaeosciurus*, rodents, Sciuridae

## Abstract

Correlations between morphology and lifestyle of extant taxa are useful for predicting lifestyles of extinct relatives. Here, we infer the locomotor behaviour of *Palaeosciurus goti* from the middle Oligocene and *Palaeosciurus feignouxi* from the lower Miocene of France using their femoral morphology and different machine learning methods. We used two ways to operationalize morphology, in the form of a geometric morphometric shape dataset and a multivariate dataset of 11 femoral traits. The predictive models were built and tested using more than half (180) of the extant species of squirrel relatives. Both traditional models such as linear discriminant analysis and more sophisticated models like neural networks had the greatest predictive power. However, the predictive power also depended on the operationalization and the femoral traits used to build the model. We also found that predictive power tended to improve with increasing body size. Contrary to previous suggestions, the older species, *P. goti*, was most likely arboreal, whereas *P. feignouxi* was more likely terrestrial. This provides further evidence that arboreality was already the most common locomotor ecology among the earliest squirrels, while a predominantly terrestrial locomotor behaviour evolved shortly afterwards, before the vast establishment of grasslands in Europe.

## 1. Introduction

Fossils are crucial for the elucidation of the evolution of biodiversity. However, as the fossil record leaves only traces of morphology, speculation remains about how extinct organisms once lived, moved and interacted with their environment. Quantitative ecomorphological analyses of extant species can reveal correlations between hard tissue structures (e.g. skeletal traits) and lifestyle, which can be used to predict the lifestyles of extinct species based on their morphology [1–3]. In turn, accurately inferring the lifestyles of extinct species can foster our understanding of past environments (e.g. [4]) and refine reconstructions of past selective regimes in order to infer the tempo of adaptive evolution (e.g. [5]). A large proportion of extant and extinct mammals consists of small, rodent-sized species [6,7] and the oldest known mammal fossils were also mouse-sized (e.g. [8,9]). Therefore, the ability to reliably reconstruct the lifestyle of small fossil species is pivotal to our understanding of mammalian evolution. However, lifestyle inferences from fossil specimens of small-sized mammals are largely problematic due to the fragile nature of their skeletal elements, which are often poorly preserved. In most cases, only dental material is preserved, while postcranial elements are usually absent or sparse and often difficult to assign to a specific taxon. Reconstructing locomotor ecology has therefore proved more challenging than feeding ecology.

Among small mammals, squirrels (family Sciuridae) constitute an insightful example. Sciuridae is one of the most species-rich and ecologically diverse families both in terms of locomotion and diet [10]. Their extant representatives exhibit a substantial locomotor diversity, ranging from arboreal to terrestrial and gliding forms of locomotion [11]. However, the evolution and diversification of these locomotor types remain poorly understood. Most of the squirrel fossil record lacks postcranial elements, while skull and especially dental features are abundant and have often been used to make predictions about habitat, lifestyle and, implicitly, locomotor ecology indirectly from taxonomic affinities (see [12] and references therein). This practice was criticized by Thorington *et al*. [13], who emphasized that the postcranium is far more indicative of squirrel locomotor behaviours and that, while dental features correlate with diet, diet is highly variable within Sciuridae independently of locomotor habits. The best-preserved fossil with a nearly complete skeleton belongs to *Douglassciurus jeffersoni* [14], one of the oldest known squirrel species from the late Eocene of North America. *Douglassciurus jeffersoni* is so similar to extant arboreal squirrels of the genus *Sciurus* that the latter has been labelled a ‘living fossil’ for demonstrating a long history of morphological stasis [14]. This view has been further strengthened by the study of postcranial elements of the extinct genus *Protosciurus* from the early to late Oligocene of North America, sharing many similarities with *Sciurus* [15]. Ancestral lifestyle reconstructions based on molecular phylogenies of extant species provide further evidence that the most recent common ancestor of squirrels was arboreal [16–18].

**Figure 1.**
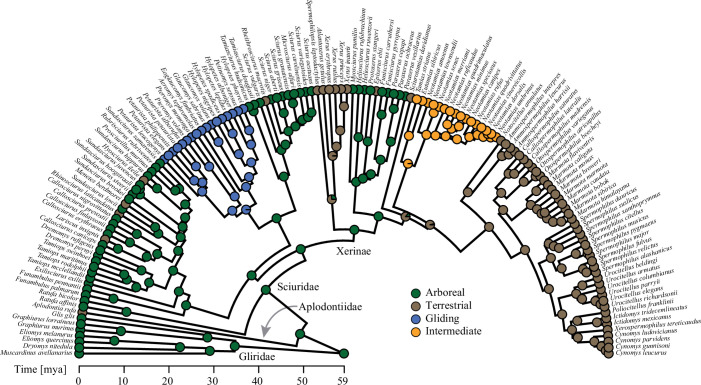
Phylogeny of Sciuromorpha. The pie charts represent the posterior probabilities of the locomotor categories at each node. Ma, million years ago.

While there is large support for an arboreal ancestor of squirrels, the evolution and diversification of other locomotor types remain uncertain, especially for the emergence of the terrestrial forms of the diverse clade of Xerinae (figure 1, [12]). *Palaeosciurus goti* from the Oligocene of France is one of the earliest terrestrial fossil squirrels based on postcranial evidence [19]. For example, the lengths of its stylopodia relative to the combined length of stylopodia and zeugopodia were large and more similar to terrestrial than to arboreal squirrels (see [20]). Qualitative limb bone comparisons with the tree squirrel *Sciurus vulgaris* as well as cranial characters like the inclination of the zygomatic plate in relation to the basicranial plane further supported the terrestrial lifestyle hypothesis [19,20]. *Palaeosciurus feignouxi*, a younger species of this genus from the early Miocene was perhaps arboreal according to Vianey-Liaud [19], as its skeletal features were more similar to *S. vulgaris* and other tree squirrels (see [20,21]). However, some limitations to these inferences exist. As stated above, cranial measurements might be problematic for the prediction of locomotor behaviour [13]. Furthermore, the limb bone ratios provided by Bryant [20] display some overlap between arboreal and terrestrial species. Finally, many features were compared on a qualitative basis and could provide deeper insight when put into a rigorous quantitative framework. Besides *Palaeosciurus*, more than 20 terrestrial xerine genera are known from the Oligocene onwards [12]. This diversification was linked to the global cooling and the spreading of grasslands during the Miocene [12,22]. However, contrary to *Palaeosciurus*, the majority of the younger fossil genera lack postcranial material, which prevents any accurate inference of their locomotor behaviours.

In this study, we quantitatively reassess the locomotor ecology of *P. goti* and *P. feignouxi* based on their femoral morphology, as certain features of this skeletal element have been shown to bear a strong locomotor ecology signal [18]. We compare them with an extensive interspecific dataset covering more than half of the extant species within Sciuridae as well as their closest relatives (Aplodontiidae and Gliridae, altogether forming the taxon Sciuromorpha; [6]). While predictions of locomotor ecology are commonly performed using a single classification technique (usually linear discriminant analysis; e.g. [3,23–25]), the rich toolkit of machine learning classification techniques enables us to determine the best possible method(s) to achieve the highest predictive power for a given dataset. Only a few comparable studies have leveraged these techniques [26–28], partly demonstrating that more sophisticated algorithms, such as neural networks, have the potential to outperform more classical techniques [28]. Here, we compare seven classification models applied to two datasets with different ways to operationalize femoral morphology. The first is a geometric morphometric dataset, and the second is a combination of univariate traits (which we refer to as the shape and the multivariate dataset, respectively, from here on to avoid confusion). Geometric morphometrics captures the shape in a rigorous manner but might also capture morphological noise that impairs discrimination between locomotor types. Selected measurements of functionally relevant features, on the other hand, might better capture the adaptive signal in the morphology and are generally more practical when fossils are damaged. We also assess the effect of body size on predictive power. Furthermore, we evaluate the importance of different femoral traits for the accuracy of making locomotor ecology predictions, as missing traits in fossils could reduce the predictability of locomotor ecology in future studies. Finally, we consider three large intraspecific samples to assess the reliability of predictions based on a few fossil specimens, which is a common problem for many extinct species.

## 2. Material and methods

### (a) Data sample

We included 240 specimens from 180 of the approximately 300 extant species within Sciuromorpha (electronic supplementary material, table S1), all of them sampled for previous studies [18,29]. We used intraspecific samples of two arboreal (*Sciurus carolinensis* and *Tamiasciurus hudsonicus*, with 22 and 18 specimens, respectively) and one terrestrial species (*Marmota monax* with 23 specimens; electronic supplementary material, table S1). These were selected due to their large geographic distributions and many subspecies [11], and because of their abundance in scientific collections. We obtained specimens from as many different locations as possible and included both sexes evenly. We included three specimens of *Aplodontia rufa*, the only extant species of Aplodontiidae. For all other 176 extant species, one specimen was sampled with no consideration of sex or side. The extinct species were *P. goti* (one specimen) from the French Quercy locality of Mas de Got (lower Oligocene) and *P. feignouxi* (six specimens) from the French locality of Saint-Gérand-le-Puy (lower Miocene; electronic supplementary material, table S1, figure S1).

### (b) Surface scan acquisition, landmarking and shape extraction

The femora were digitized using surface or microcomputed tomography (µCT) scanners from various institutions. Left femora were mirrored in Geomagic Studio 2013.0.2 (3D Systems, Rock Hill, SC, USA) so that the dataset only contained right ones. We placed traditional and sliding semilandmarks [30–32] in IDAV Landmark v. 3.0 [33]. All downstream analyses were run in R v. 4.4.1 [34]. The functions placePatch and slider3d from the package Morpho [35] were used to determine the positions of semilandmarks, resulting in 192 final three-dimensional landmarks. See electronic supplementary material, S1 for detailed information on the scanners used as well as the landmarking and shape extraction procedures.

### (c) Acquisition of univariate traits and body size

**Figure 2.**
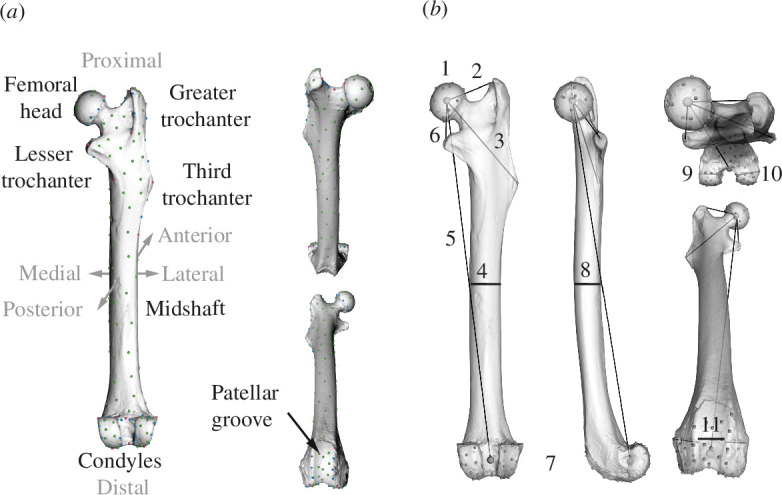
Landmarks and univariate traits acquired from the femora. The mirrored scan of the femur from *Palaeosciurus goti* is depicted. (*a*) Morphology and topology of the femur and landmarks used for shape analysis. Red: fixed landmarks. Blue: curve sliding semilandmarks. Green: surface sliding semilandmarks. (*b*) Analysed femoral traits. 1: centroid size of the femoral head landmarks. 2: gluteus medius in-lever. 3: gluteus superficialis in-lever. 4: mediolateral midshaft diameter. 5: effective femoral length. 6: iliopsoas in-lever. 7: centroid size of the distal condyles and the patellar groove landmarks. 8: anteroposterior midshaft diameter. 9: medial condyle width. 10: lateral condyle width. 11: patellar groove width.

We used the univariate traits from Wölfer *et al*. [18], including effective femoral length, osteological in-levers of three muscles, as well as traits reflecting the robustness or size of various substructures (figure 2). These were either extracted from landmark information or measured on the surface scans (see [18]). Our body size proxy was defined as the geometric mean of all 11 univariate traits (11th root of the product of the 11 univariate measurements examined here; see [3]).

### (d) Locomotor ecology of extant species

The extant species were categorized into arboreal, terrestrial, intermediate and gliding according to information retrieved from the literature [11,36]. Arboreal species are agile climbers, sometimes foraging on the ground. Terrestrial species live on the ground and rarely climb, most of them digging underground burrows. Species of the intermediate category belong to the tribe Tamiini (chipmunks). They are generalists that climb well but also live on the ground and dig burrows. Gliding species are arboreal but possess a patagium to glide from tree to tree.

Locomotor regimes were reconstructed along a phylogeny to assist the interpretation of predictions of fossil locomotor ecology. We used the phylogeny from Wölfer *et al*. [18], who fused the sciurid phylogeny from Zelditch *et al*. [37] with taxa from the TimeTree database [38]. This phylogeny was pruned to 142 species that were also included in our morphological dataset. We used stochastic character mapping [39,40] implemented in the make.simmap function of the package ‘phytools’ [41] to reconstruct ancestral locomotor types, constraining the transition rate matrix to all rates being different. One thousand character maps were generated to create posterior probabilities of the four locomotor categories at each internal node (figure 1).

### (e) Morphospace analysis

We conducted a principal component analysis (PCA) with all specimens on the Procrustes coordinates using the gm.prcomp function from the geomorph package [42] and on the multivariate dataset using the prcomp function. We plotted the principal components (PCs) with a variance above 5% to visualize major patterns related to the clustering of locomotor ecology and the morphospace occupation of intraspecific samples. The univariate traits were transformed using the natural logarithm and centred and scaled before PCA. The intraspecific variability of *M. monax*, *S. carolinensis* and *T. hudsonicus* was considerably large when compared with the interspecific variability (see Results). Therefore, we included all of their specimens as separate observations in the machine learning models to maximize sample size. We compared the disparity of *P. feignouxi* with each of the three intraspecific samples using convex hull volumes (Quickhull algorithm [43] implemented in the convhulln function of the geometry package [44]) on the first five PCs (limited by the sample size of *P. feignouxi*). We also calculated the sum of variances using the var function across all PCs. The volumes and variances of *P. feignouxi* were then transformed into percentages of the values obtained from the respective extant intraspecific sample.

### (f) Machine learning approach

We used the caret package [45] and the MLmetrics package [46] to streamline the comparison of the predictive power of different machine learning models. The multivariate dataset was transformed using the natural logarithm for all further analyses. Both extant datasets were split in the same way by locomotor category into a training set of approximately 70% to build the models and a test set of 30% to test the predictive power of the models.

Body size is an important determinant of femoral morphology besides locomotor ecology and thus may complicate discrimination between locomotor categories. In Sciuromorpha, the specific allometric scaling relationship of various femoral traits depends on the respective locomotor category, meaning that both effects are not additive and cannot be simply subtracted from another [18]. To test how body size affects our predictions, we trained and tested all predictive models with and without size-corrected data (see below). The correlations between all univariate traits in the dataset without size correction were very high (analysed using the ggpairs function of the GGally package [47]), which is also reflected in the PCA results (see below). However, we did not account for multicollinearity, as it is not considered a severe modelling problem for prediction (as opposed to explanation; see [48] and references therein). Size correction with the training data was performed by obtaining residuals from a multivariate regression (lm function) on the geometric mean. We used ordinary least squares (OLS) instead of phylogenetic generalized least squares (PGLS) regressions because the femoral morphology in Sciuromorpha is barely affected by phylogenetic inertia [18]. The residuals of the test set and fossil data were then obtained by first computing their predicted trait values from their geometric mean based on the regression coefficients from the training data and then subtracting these values from their actual values.

We applied a PCA to the training shape datasets for the purpose of variable reduction. In both the original and size-corrected shape datasets, the first 13 PCs accounted for 99.5% of the shape variance and were retained for further analysis. The test and fossil datasets were then projected into the PC space built by the training dataset.

Seven different models, k-nearest neighbours (kNN), linear discriminant analysis (LDA), multinomial logistic regression (MLR), neural network (NN), random forests (RF) and support vector machine with either a linear (SVM_l_) or a radial kernel (SVM_r_), were fitted to all training datasets using the train function of the caret package (similar models were used previously by Püschel *et al*. [26,27] and Ballell *et al*. [28]).

**Figure 3.**
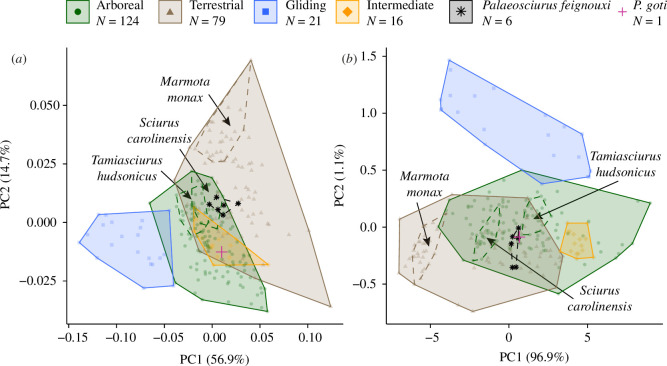
First two principal components of the analysed datasets. (*a*) Shape dataset. (*b*) Multivariate dataset.

We always used the mean sensitivity, i.e. the proportion of correct classifications per locomotor category averaged across all four categories, as a measure of predictive power. To optimize the model hyperparameters, we used five rounds of cross-validation with 10 folds each, always resampling by locomotor category and using the same splitting for all models. We used upsampling on our training data during the cross-validation procedure, so that all classes in each fold had the same sample size to prevent majority classes from dominating model building. Since the sample sizes varied widely (figure 3), this resulted in many random replications for the minority classes (gliding and intermediate). We compared these models with predictive models constructed without upsampling (using the datasets without size-corrected data) to see if class imbalance affects model assessment and prediction of fossil locomotor ecologies. For each model, the hyperparameter value with the largest mean sensitivity during model building was selected to check the mean sensitivity of each final model against the test set. See electronic supplementary material, S2 and table S2 for more details on the modelling process and the final hyperparameter values. Lastly, we computed the probability of each of the seven fossils belonging each locomotor category.

We further analysed the dependency of the mean sensitivity on body size using the test data and the models built with upsampling from both shape and multivariate datasets without size correction. We used each model with the best predictive performance to predict the locomotor categories. Then, a binary misclassification variable was generated with ‘1’ indicating a false and ‘0’ indicating a correct prediction of a specimen when comparing its predicted with its actual locomotor category. The glm function was used to regress this misclassification variable on the geometric mean using a binomial distribution with a logit link function. The margins function from the margins package [49] was used to calculate the average marginal effect, i.e. the average rate of change of the predicted probability of being misclassified with increasing body size. The cplot function was used to graph the predicted probability of being misclassified against the geometric mean and we used the predict function to obtain the probability of misclassification for the fossil specimens.

**Figure 4.**
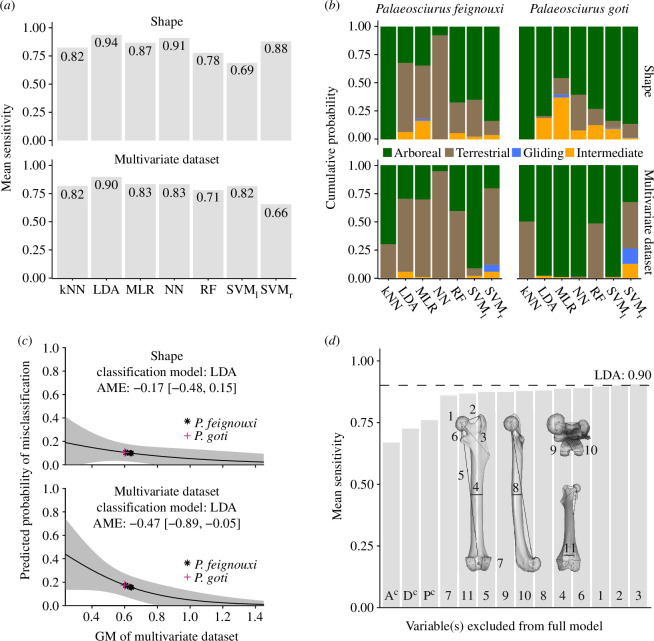
Results for the prediction of locomotor categories. Results are shown for the models built with upsampling and without size correction. (*a*) Mean sensitivity of different models built by the training datasets. (*b*) Probabilities of belonging to each locomotor category for the two studied fossil species (probabilities of the six specimens of *P. feignouxi* are averaged). (*c*) Predicted probability of misclassification versus body size proxy (i.e. the geometric mean (GM) of the 11 femoral traits). The shaded area represents the 95% CI, and the average marginal effect (AME) is shown. (*d*) Variable importance of femoral traits in terms of decrease of mean sensitivity when one or more variables are removed from the best model (see panel a). A^c^: complement of the aspect ratio was removed, i.e. variables 4, 5 and 8 were retained. D^c^: complement of the distal traits was removed, i.e. variables 7, 9, 10 and 11 were retained. P^c^: complement of the proximal traits was removed, i.e. variables 1, 2, 3 and 6 were retained. kNN, k-nearest neighbours; LDA, linear discriminant analysis; MLR, multinomial logistic regression; NN, neural network; RF, random forests; SVM_l_, support vector machine with a linear kernel; SVM_r_, support vector machine with a radial kernel.

Finally, we assessed the importance of the 11 femoral traits for locomotor category prediction. Again, we used the best model built with upsampling and without size correction. After removing one variable at a time, we repeated the model fitting and computed the mean sensitivity. The greater its decrease as compared with the full model, the more important the variable is for predicting locomotor ecology. Because fossil femora are often broken in half, we also tested models only built with the proximal or distal features. Both datasets included four features, none containing femoral length and the midshaft diameters as these would not be available in these cases (figure 4). Finally, we assessed the predictive power by considering only femoral length and the two midshaft diameters (called the femoral aspect ratio) since these have been used previously to assess femoral robustness as an indicator for the degree of terrestriality in squirrels (e.g. [50–52]).

## 3. Results

### (a) Morphospace description

The first two PCs of the shape dataset represented *ca* 72% of the variance (figure 3). The gliding species are well separated from the rest along PC1. Arboreal and terrestrial species partly overlap. This overlapping area is also occupied by the intermediate category (i.e. Tamiini). The third and fourth PCs contained 7% and 5% of the variance, respectively, and did not contribute to the separation of the locomotor categories (electronic supplementary material, figure S4). The six specimens of *P. feignouxi* occupy a relatively small region of the morphospace and are located closer to the centre of the convex hull of the terrestrial species and the margin of the hull of the arboreal species (figure 3). *Palaeosciurus goti*, on the contrary, is located closer to the centre of the arboreal species but also the intermediate ones. The hulls of the three extant intraspecific samples exceed that of *P. feignouxi*. The convex hull volume of the *P. feignouxi* measured to be 0.18%, 0.74% and 0.07% of the convex hull volumes of *S. carolinensis*, *T. hudsonicus* and *M. monax*, respectively. In terms of summed variances, these percentages were 74%, 92% and 48%, respectively.

The first two PCs of the multivariate dataset accounted for approximately 98% of the shape variance (figure 3; all Pearson’s correlation coefficients were above 0.9; electronic supplementary material, figure S5). The gliding species are again well separated from the rest but along PC2. The arboreal and terrestrial species appear to overlap more compared with the shape dataset. In contrast, the species belonging to the intermediate category are located in the morphospace occupied by the arboreal species. The fossil specimens occupy a region where arboreal and terrestrial species overlap. The convex hull volume of the *P. feignouxi* measured 0.47%, 0.44% and 0.09%, and the summed variances were 46%, 47% and 38% in relation to *S. carolinensis*, *T. hudsonicus* and *M. monax*, respectively.

### (b) Predictive power of the tested models

The mean sensitivity varied between the models and between the shape and multivariate datasets. In the models built with upsampling and without size-corrected data, it ranged from 0.69 to 0.94 in the shape dataset and from 0.66 to 0.90 in the multivariate dataset (figure 4*a*). Between the two datasets, the mean sensitivity of the same model varied up to a difference of 0.22 (SVM_r_). LDA had the highest mean sensitivity in both datasets, 0.94 for the shape and 0.90 for the multivariate dataset. For the shape dataset, NN was second best (0.91), and MLR and SVM_r_ were also powerful in comparison (0.87–0.88, respectively), while for the multivariate dataset, kNN, MLR, NN and SVM_l_ ranked behind LDA, all having similar mean sensitivity values (0.82–0.83).

In the models built without upsampling, similar trends were present despite minor differences. For example, for the shape dataset, LDA and NN also performed best, and the differences in mean sensitivity between all seven models were very similar (compare figure 4*a* and electronic supplementary material, figure S6A). Regarding the multivariate dataset, NN was the most powerful model with a mean sensitivity of 0.95, but LDA and MLR were still among the best models (0.86 and 0.89, respectively; electronic supplementary material, figure S6A).

When comparing the models built with size correction to those without it, the mean sensitivity decreased for almost all models (compare electronic supplementary material S6C and figure 4*c*). For example, the mean sensitivity of the NN and LDA models decreased by 0.9 and 0.16 (shape dataset) and by 0.17 and 0.16 (multivariate dataset), respectively. The only models that improved in mean sensitivity were RF (by a difference of 0.15) and SVM_r_ (by 0.06), both built using the multivariate dataset (electronic supplementary material, figure S6C).

### (c) Locomotor ecology prediction of fossils

Focusing on the models built with upsampling and without size-corrected data, for *P. feignouxi*, the probabilities of belonging to a particular locomotor category depended largely on the model but also partly on the dataset (figure 4*b* and electronic supplementary material, S7). According to the most powerful models of both datasets (LDA, NN and MLR), the terrestrial category was the most probable one (figure 4*b*), although the probability of the arboreal class was high in a few individual specimens according to the LDA and MLR models (electronic supplementary material, figure S7). The SVM_r_ model (third best) and some other models with intermediate or low predictive performance returned a high average probability for the arboreal category (figure 4*b*). All of the seven models assigned *P. feignouxi* a very low probability of being gliding or intermediate in its locomotor behaviour (figure 4*b* and electronic supplementary material, S7).

For *P. goti*, 10 out of 14 models assigned the highest probability to the arboreal category, including those models with the best predictive power (figure 4*b*). Three models assigned the highest probabilities to both the arboreal and terrestrial categories. Similar to *P. feignouxi*, the probability of *P. goti* belonging to the gliding or intermediate locomotor category was very low, although the MLR model of the multivariate dataset assigned about equal probabilities to the arboreal and intermediate classes (figure 4*b*).

The models built without upsampling yielded almost identical probability distributions compared with the models built with upsampling (electronic supplementary material, figure S6B). However, those built with the size-corrected data showed different patterns depending on the dataset. For the multivariate dataset, the models also yielded very similar predictions, whereas the models built with the shape dataset tended to yield an increased probability for the intermediate category (electronic supplementary material, figure S6D).

### (d) Predictive power versus body size

The predicted probability of misclassification decreased with body size (figure 4*c*). This effect was weaker in the shape dataset and it could not be excluded that body size had no effect on the probability of misclassification, as the confidence intervals of the average marginal effects included the value ‘0’ ([−0.48, 0.15] as compared with the multivariate dataset with [−0.89, −0.05]; figure 4*c*). The geometric mean of fossil specimens ranged from 0.6 to 0.64, resulting in predicted misclassification probabilities ranging from 0.1 to 0.11 (shape) and 0.16 to 0.18 (multivariate dataset).

### (e) Variable importance

The largest decrease in mean sensitivity (from 0.90 to 0.67) occurred when only the femoral length and the midshaft diameters, which determine the femoral aspect ratio/robustness, were retained (figure 4*d*). The mean sensitivity decreased to 0.73 when only the proximal traits and to 0.76 when only the distal traits were retained. The removal of any single variable had a much smaller effect on the mean sensitivity, with the smallest value being 0.86 for the centroid size of the condyles. No single variable could be considered outstandingly important, as the mean sensitivity increased uniformly from the most to the least important variable (figure 4*d*).

## 4. Discussion

### (a) Influence of machine learning method and operationalization on ecomorphological predictions

We first compared the predictive performance of different machine learning methods and different operationalizations (shape and multivariate datasets) to determine the most powerful model for predicting locomotor ecologies. Previous comparable studies used either shape or von Mises stresses from finite element models as predictors of either locomotor ecology from primate talar morphology [26,27] or feeding ecology from sauropsid tooth morphology [28]. Comparisons are limited because these studies differed from ours in their performance measures (using accuracy and Cohen’s kappa), in not accounting for class imbalance and in using only size-corrected data to build their models. In any case, the predictive performance varied widely between the machine learning algorithms used here (by up to about 25%) and also in Püschel *et al.* [26,27] and Ballell *et al.* [28], indicating that classification techniques in general vary widely in their predictive ability when applied to ecomorphological datasets. Testing different machine learning techniques to maximize predictive power has become a staple in many biological disciplines (e.g. [53–57]). We recommend that this should also become standard practice when predicting ecology from morphology.

Testing different classification models raises the question of whether one or a few are best suited for lifestyle predictions from morphological data since dozens of models are available nowadays (e.g. the modelLookup function from the R package caret v. 6.0–94 lists 191 models for classification). The choice of a particular subset may be a matter of subjective preference, the need to keep computational complexity within reasonable limits, or tradition in the research field. For example, LDA, a comparatively simple linear classification technique, seems to have been the most widely used method for fossil lifestyle predictions in the past (e.g. [3,23–25,58,59]). LDA proved to be the best model for our datasets and among the best models in Püschel *et al*. [26,27]. However, LDA was the second worst model in Ballell *et al*. [28], suggesting that it should not be considered the optimal model *per se* for making predictions and that testing alternatives is recommended.

NNs are increasingly being applied in palaeontology to predict taxonomic relationships from fossil images [60–63]. It was the best performing model in the study by Ballell *et al*. [28] as well as the best in our study (without upsampling) or the second best (with upsampling), providing evidence that NNs are a powerful tool for ecomorphological predictions besides traditional methods, like LDA. The performance of the NN model in Ballell *et al*. [28] and here is surprising, as the large number of parameters associated with NNs and the relatively small sample sizes typically lead to overfitting. Alwosheel *et al*. [64] recommended a sample size of 50 times the number of weights in the NN model. The NN model for our multivariate dataset was far from meeting this recommendation by including 100 weights and a training data sample size of 170 observations. It is questionable whether the requirements for a NN suggested by Alwosheel *et al*. [64] are achievable, as sample size in macroevolution is limited by the number of available species/specimens and the taxon under consideration. Further model comparisons are needed to determine whether certain machine learning classification methods such as NNs are generally powerful in predicting ecomorphological patterns across taxa despite such sample size limitations.

What has not been discussed is how phylogenetic non-independence affects the predictive power of the plethora of models available. Phylogenetic non-independence is typically an autocorrelation problem associated with residuals in regression models that are used to infer adaptive relationships between phenotypic and/or ecological traits [65,66]. Future studies need to focus on how this type of autocorrelation affects prediction and how it should be accounted for in different types of machine learning methods, as it has been discussed for spatial data, which are often confronted with spatial autocorrelation (e.g. [67]).

We found that, in addition to the choice of statistical model, the operationalization of the morphological element under consideration can also determine the predictive power, the shape dataset mostly outperforming the multivariate dataset. Similarly, Püschel *et al*. [26,27] found that shape always outperformed von Mises stresses for their dataset. However, in the study by Ballell *et al*. [28], which also used shape and von Mises stresses, no dataset appeared to be superior across different models. Still, these findings indicate that quantifying shape rigorously can benefit ecomorphological predictions despite introducing some noise. However, further research is recommended to assess whether certain forms of operationalization are generally more powerful in predicting ecology from morphology.

### (b) Most probable locomotor behaviours differ between *P. feignouxi* and *P. goti*

Reconstructing locomotor ecology from fossils of small mammals is challenging due to the paucity of postcranial material from which to make ecomorphological comparisons with extant species. Here we reassessed the locomotor behaviour of two of the oldest squirrel species on the basis of their femoral morphology. While misclassification of locomotor category tended to increase with decreasing body size, the probability of misclassifying the relatively small *Palaeosciurus* specimens was still predicted to be below 0.2 (figure 4*c*), making body size a less critical factor in predicting their locomotion. According to all models, *P. feignouxi* and *P. goti* were unlikely to be gliding rodents. This is consistent with evidence from molecular phylogenetics suggesting that gliding squirrels split off from their arboreal relatives only during the early Miocene [22] and is supported by the oldest postcranial evidence for a gliding squirrel, i.e. the mid-Miocene species *Miopetaurista neogrivensis* [68].

The fact that the most likely locomotor ecology of *P. goti* was arboreal according to almost all models of both datasets contradicts a previous attempt to infer a terrestrial ecology [19] based on limb proportions and the zygomatic plate orientation. To our knowledge, *P. goti* is the only Oligocene fossil that was hypothesized to be terrestrial on the basis of postcranial morphology. According to a literature review conducted by Ge *et al*. [12], Oligocene fossils from the genera *Protospermophilus*, *Miospermophilus*, *Nototamias*, *Tamias*, *Heteroxerus* and *Atlantoxerus* were also considered terrestrial. However, our review of literature shows that a terrestrial ecology has always been inferred from the taxonomic affinity of the fossils with extant species according to cranial, mandibular and dental characters (e.g. [69–75]). As discussed by Thorington *et al*. [13], such characters may be far less reliable than postcranial characters for discriminating locomotor behaviours, but postcranial material is lacking for these specimens as far as we know. Consequently, our prediction of an arboreal locomotor ecology for *P. goti* may indicate that a terrestrial locomotor behaviour did not evolve in squirrels before the Miocene.

According to the majority of the best models for each dataset, the terrestrial category was more likely for *P. feignouxi*, whereas it was previously reconstructed as potentially more arboreal than *P. goti* on the basis of limb proportions [19]. If true, but more evidence is needed, this would imply that squirrels within the genus *Palaeosciurus* experienced selective pressure to adopt a terrestrial locomotor behaviour prior to the widespread occurrence of open grasslands in western Europe during the middle Miocene (e.g. [76]). The evolution of terrestriality within a predominantly arboreal habitat is plausible. Squirrel species evolved independently a terrestrial or intermediate locomotor behaviour (in terms of ground-dwelling) multiple times within approximately the last 10 Myr (figure 1, [17]), while remaining in an arboreal habitat like their close relatives.

While we have strong support for these predictions, sampling more specimens is likely to increase the uncertainty, firstly because all *Palaeosciurus* specimens fell in or close to a morphospace region where arboreal and terrestrial species overlapped (figure 3), and secondly because we could demonstrate that the three intraspecific datasets of around 20 specimens each exceeded the morphological variability of the six specimens sampled for *P. feignouxi*. Analysis of other postcranial elements may further improve the clustering of locomotor behaviours and hence, their prediction. For example, Rickman *et al*. [77] showed that certain humeral features distinguish terrestrial from arboreal squirrels. Tail length is another strong indicator of locomotor behaviour in squirrels [78], but complete sequences of caudal vertebrae are typically not available in the fossil record [79].

### (c) Complicating factors for ecological predictions in squirrels and other small mammals

Body size can become a complicating factor in discriminating locomotor behaviours on the basis of morphological traits due to allometric effects [26], which might also vary between locomotor categories [18]. Jenkins [80] argued that small ground- and tree-dwelling mammals face similar biomechanical challenges, as both have to move over uneven terrain relative to their body size. The small tree squirrel *Tamiops swinhoei*, for example, displays very similar kinetics and kinematics when running on both flat and branch-like supports [81]. However, it has been shown that the effect of size on the discriminability between tree- and ground-dwelling mammals depends on the morphological trait under study [82]. We found that the probability of misclassification in Sciuromorpha based on femur morphology tended to increase with decreasing body size. This might be related to the decrease in predictive performance of most of our models after removing the effect of body size from the data. It may indicate that the size of a skeletal element is an important intrinsic morphological feature that can improve the discrimination between ecological categories, especially in the presence of interaction effects between size and ecology (e.g. [18]). In addition, Weaver & Grossnickle [82] found that discrimination between small tree- and ground-dwelling mammals increased when femoral length was used but tended to decrease when distal condyle width was used. This suggests that the effect of body size on misclassification may depend on the traits under consideration, which may be important to consider when making ecological predictions for fossil specimens of small mammals.

The effect of body size on misclassification observed here was probably driven by the arboreal, fossorial and gliding categories, which had a large body size range in terms of the geometric mean of the femoral traits (0.21–1.17, 0.42–1.62 and 0.34–1.15, respectively). In comparison, the intermediate locomotor category was only represented by the small chipmunk species in our dataset, whose geometric means ranged from 0.33 to 0.43. Consequently, the intermediate (i.e. mixed arboreal/terrestrial) locomotor ecology is probably the most difficult one to identify in fossil squirrels. Large fossil species with such a locomotor behaviour are unlikely to be identifiable by their femoral morphology due to the lack of representation in extant species. However, after removing the effect of size from the shape dataset, the probability that both *Palaeosciurus* species belonged to the intermediate category increased immensely for the majority of models. Perhaps, since *Palaeosciurus* is about twice as large as Tamiini, both taxa differ in their femoral shape only because of an allometric scaling effect that was removed after size correction, although this did not appear to be the case for the multivariate dataset. If this is true, *Palaeosciurus* may represent a genus at the transition from an arboreal locomotor ecology as assumed for *Douglassciurus* [14] to a predominantly terrestrial ecology as observed in Xerinae [12]. In any case, the decrease in predictive performance of these models that were built using the size-corrected data cautions against overinterpreting their predictions for the time being.

Another complicating factor is the assignment and definition of locomotor categories. The discrepancy between the continuous diversity of locomotor behaviours in living species and the operational categories used in ecomorphological studies has constituted a long-standing issue [83]. In squirrels, three to four ecotypes have been recognized [84] and repeatedly used for ecomorphological comparisons (e.g. [50,77,85–87]). We also referred to this classification scheme by using the categories arboreal, intermediate, terrestrial and gliding. The tribe Tamiini is considered intermediate in between arboreal and terrestrial squirrels in its ecology and skeletal characteristics [84]. According to our femoral shape dataset, it indeed occupies the morphospace region where arboreal and terrestrial squirrels overlap (figure 3). However, when using the univariate traits, Tamiini appeared to fall into the arboreal category (figure 3). In this case, it may be justified to assume that the arboreal loading regime exerts a higher selective pressure on the femur in Tamiini than the terrestrial loading regime (e.g. [18]). This also means that we would not be able to distinguish between intermediate and arboreal squirrels in the fossil record using these traits. Indeed, many squirrels that are typically classified as arboreal also come down to the ground to collect and hoard food [11]. Future predictions of the locomotor ecology of extinct species might benefit from a more refined approach that allows for finer-grained locomotor categories or the incorporation of multivariate ecology as demonstrated, for example, by Wisniewski *et al.* [88]. We also cannot exclude the possibility that extinct squirrels had a locomotor repertoire not represented by their living relatives (e.g. aquatic or cursorial locomotion as found in other rodent taxa [36]).

Finally, the number and preservation of skeletal elements limit the predictability of ecological traits. In the case of our analysis of the femur of squirrels, missing a single trait is less critical, but missing either the proximal or distal region can drastically affect the discriminability between locomotor categories in Sciuromorpha. This has implications for prediction for fossils where only half of the femur is preserved. Previous works have focused on femoral robustness as an indicator for the degree of terrestriality in squirrels by evaluating the femoral aspect ratio, i.e. relating femoral length to femoral thickness [50–52]. The robustness of long limb bones is a similarly important discriminator for identifying gliding squirrels in the fossil record [68], and it is also an indicator of different locomotor ecologies in rodents in general [59]. We demonstrated that just considering the femoral aspect ratio leads to a severe decrease in predictive power as compared with using the complete multivariate dataset. More studies on other skeletal elements are necessary to judge if this pattern is generalizable to other long bones, but our study suggests that including as many traits as possible for the purpose of ecological predictions can be beneficial for maximizing predictive power.

## 5. Conclusion

Predicting the locomotor behaviour in fossils of small mammals such as squirrels can be challenging due to size effects on ecomorphological discriminability, limited sample sizes and availability of postcranial material and the difficulty in accounting for the continuity in the behavioural spectrum of a species. By leveraging all available traits into multivariate datasets and using the rich toolkit of machine learning methods, we can maximize our confidence in the predictive performance of locomotor ecology reconstructions in the fossil record. In our case, this led to the reclassification of *P. goti* as arboreal, a species previously considered to be the oldest terrestrial squirrel. The fact that *P. feignouxi* was most likely terrestrial suggests that squirrels rapidly evolved this locomotor behaviour, which is still observed in extant taxa. We demonstrated the importance of testing different machine learning methods, since inferences may depend on the choice of model. In such cases, the selection of the best model(s) becomes pivotal, which may depend on the dataset at hand in terms of traits and operationalization. We suggest that model comparison becomes a standard procedure in palaeontological studies concerned with predicting ecology from fossils (see [26–28]), as it already is in many other biological disciplines.

## Data Availability

Data and scripts to reproduce the data analysis are available at [89]. CT data of the *Palaeosciurus* specimens are available on MorphoSource (https://doi.org/10.17602/M2/M664446, https://doi.org/10.17602/M2/M664983, https://doi.org/10.17602/M2/M664993, https://doi.org/10.17602/M2/M664398, https://doi.org/10.17602/M2/M664387, https://doi.org/10.17602/M2/M664359, https://doi.org/10.17602/M2/M664324) and the surface scans derived from these CT scans are published on MorphoMuseuM [90]. Supplementary material is available online [91].
